# Investigating the association between occupational exposure to asbestos and ovarian carcinoma: results from a pilot study in Germany

**DOI:** 10.1186/s12889-019-7590-7

**Published:** 2019-10-22

**Authors:** Zara Rajput, Kurt Georg Hering, Thomas Kraus, Andrea Tannapfel, Günter Sonnenschein, Alexandra Centmayer, Katja Radon, Dennis Nowak, Tobias Weinmann

**Affiliations:** 1 Institute and Clinic for Occupational, Social and Environmental Medicine, University Hospital, LMU Munich, Ziemssenstr. 1, 80336 München, Germany; 2 Department of Diagnostic Radiology, Radiooncology and Nuclear Medicine, Radiological Clinic, Miner’s Hospital, Dortmund, Germany; 3 Institute for Occupational, Social and Environmental Medicine, RWTH Aachen University, Aachen, Germany; 4 Institute for Pathology, Ruhr University Bochum, Bochum, Germany; 5 Sachverständiger Asbest, Düsseldorf, Germany; 6 Gesundheitsvorsorge (GVS), Berufsgenossenschaft Energie Textil Elektro Medienerzeugnisse (BG ETEM), Augsburg, Germany

**Keywords:** Asbestos, Occupational diseases, Ovarian neoplasms, Cancer epidemiology, Epidemiological methods

## Abstract

**Background:**

The aim of this pilot study was to assess the feasibility of a large-scale epidemiologic investigation elucidating the quantitative association between occupational exposure to asbestos and ovarian cancer in former German asbestos workers.

**Methods:**

Between December 2017 and May 2018, a random sample of one thousand insured woman registered at the health service of a German trade association as formerly occupationally exposed to asbestos were invited to participate in a pilot study. Participation included a phone interview using a standardised questionnaire. The feasibility of the project was evaluated using a priori defined criteria. They included response, number of cases, eligibility of the questionnaire data for exact estimation of asbestos fibre-years, and availability of relevant medical documentation (imaging procedures, medical reports, and histologic materials).

**Results:**

The response (17%) was clearly below the intended number of 60%. With six tumour suspects, of which two could be confirmed by medical documents, the number of cases was within the expected range of two to eleven cases. Exact asbestos fibre-year estimations could be performed for 29% of all interviewees, but only for one suspected case. Medical documentation could be collected for only few participants, while no histology reports could be obtained for all cases. Thus, only the feasibility criterion of the expected number of cases was fulfilled.

**Conclusion:**

The results of the pilot study indicate that the planned project is feasible only to a very limited extent. For further planning of the study, measures to improve recruitment of participants are necessary.

## Background

Ovarian cancer is the seventh most common cancer in women globally [1] and the fifth most frequent in Europe [2]. Moreover, it is considered the most lethal gynaecologic malignancy. According to the Cancer Incidence and Mortality Worldwide study, in 2015 there have been 152,000 deaths reported due to ovarian cancer while 239,000 new cases were registered. Therefore, scientific research has focused on trying to identify the risk factors associated with this fatal cancer in order to find ways for prevention of the disease [3, 4].

So far, many factors associated with ovarian cancer have been identified [5]. Factors that were shown to be protective are use of oral contraceptive pills (OCPs), parity, breastfeeding, and possibly physical activity [6–8]. Potential risk factors include early age at menarche and late age at menopause, smoking, endometriosis, pelvic inflammatory disease, obesity, asbestos exposure, and use of talcum powder [1, 6, 9–11]. Breast cancer genes 1 and 2 (BRCA 1 and BRCA 2) were found to be present in 10–15% of ovarian cancers [12]. Role of diet, physical exercise, and Vitamin D levels are yet to be further investigated [1].

Amongst occupational factors, asbestos is of huge importance [13, 14]. There are 125 million people in the world working in an environment with asbestos exposure and previous studies have found that 90,000 people die every year due to mesothelioma, asbestosis, or lung cancer caused by asbestos [15]. Similarly, asbestos fibers were hypothesised to induce inflammation in the epithelium of the ovaries which increases carcinogenesis [16]. Some studies detected fibers in the ovaries of women working with asbestos [17]. Consequently, a systematic review and meta-analysis observed good evidence for an association between asbestos exposure and ovarian cancer, although it could not deduce information on the dose-response relationship [18]. Hence, there is still insufficient knowledge regarding the quantitative association between asbestos exposure and ovarian cancer risk. Reliable evidence on the dose-response relationship would not only be interesting for scientific purposes, but also for potential adjustment and refinement of the occupational disease law in Germany. In addition, no studies in Germany have particularly investigated asbestos and ovarian cancer, although until its ban in 1993 asbestos use was widespread in German industry. Furthermore, based on previous knowledge, pleural thickening acts as a reliable marker for asbestos exposure, thus it would be interesting to know if it can also serve as a marker for early detection of asbestos-related ovarian cancer [19, 20]. Such knowledge would not only have high prognostic value but could also help to develop specific screening and prevention programmes.

We therefore aimed at testing the feasibility of a large-scale retrospective cohort study among women in Germany occupationally exposed to asbestos including reliable and accurate estimation of the quantitative relationship between asbestos exposure and ovarian cancer by means of a pilot study. As feasibility criteria we assessed participants’ willingness to participate (response), the number of detected cases, the eligibility of the questionnaire data for exact estimation of asbestos fibre-years (AFY), and the availability of relevant medical documentation (records from imaging procedures, medical reports, and histologic materials for tumour validation).

## Methods

### Study design and setting

Between December 2017 and May 2018, one thousand out of about 16.000 insured woman registered at the health service of a German trade association as having been occupationally exposed to asbestos were invited to participate in a pilot study. They received a postal invitation package containing an information letter on the details of the study along with a consent form as well as a return envelope to send back the informed consent form. Non-respondents were reminded up to two times. Trained professionals via telephone individually interviewed those women who gave written informed consent to participate. During the interview, a study questionnaire that incorporated validated instruments from previous studies was completed.

### Sample size

Since it can be assumed that at the time of the asbestos ban in Germany in 1993, the registered women were at least 16 years of age (as they must have been employed, otherwise they could not appear in the registry), we reckoned that the youngest members of the cohort were now at least 38 years old. Applying the incidence rate for Germany provided by the Robert Koch Institute of 29.9 cases per 100,000 persons in the age range of 35 to 84 years to our total sample of 16.000 registered women, 4.8 cases per year could be expected [21]. Hence, during a 22-year follow up there should be 106 cases of ovarian cancer in the sample due to natural rate of disease. Applying the odds ratio calculated by the above mentioned meta-analyses of 1.77 (95% CI: 1.37–2.28), around 200 cases should be expected [18]. In the pilot study, 5% of the the registered women should be included, i.e., 800 persons. Reckoning with a willingness to participate of 80%, a random sample of 1000 women was invited. Based on the above-mentioned calculations, the expected number of cases among those 800 individuals was 5 cases (95% CI: 2–11 cases).

### Assessment of the outcome

During the interview, all participants were asked if they ever had a diagnosis of ovarian cancer. In case of an affirmative answer, the interviewers asked them for the year of diagnosis and the clinic in which they were treated. As described below, we then tried to collect medical documentation and histologic materials for tumour validation.

### Exposure assessment

For assessment of asbestos exposure, the study questionnaire asked for the participants’ full occupational history including duration (start and end date), job title, tasks and activities, form of employment (full-time or part-time), and exposure to asbestos (yes/no) for each job that was performed for at least 12 months. In case of jobs containing asbestos exposure, participants answered a job-specific supplementary questionnaire. According to the specific type of job reported, interviewers selected one out of 33 job-specific questionnaires (Table 1) that were specifically tailored for collecting detailed information on various aspects of asbestos exposure such as specific materials, distance to the source of exposure, or use of personal protective equipment. Ahrens and colleagues developed those job-specific questionnaires in the 1990s specifically for retrospective assessment of occupational asbestos exposures in German study populations [22, 23]. For the purpose of the present study, we developed an additional supplementary questionnaire, which the interviewers used if the job reported by the interviewee could not be allocated explicitly to one of the 33 job-specific questionnaires. This additional questionnaire contained those aspects of asbestos exposure which the German Social Accident Insurance considers as the most important ones to calculate exposure levels in asbestos fibre-years [24]. Those were type of activity, start and end date, frequency per week, duration per day, type of premises, distance from the source of asbestos exposure, use of personal protective equipment, type of materials and type of tools used, use of asbestos at neighbouring work places, and ventilation measures. Lastly, an expert (GS) calculated individuals’ exposure levels in asbestos-fibre years using the data provided by the supplementary questionnaires. We chose AFY as our primary measure of exposure as they are the valid and applied measure of asbestos exposure in Germany’s occupational disease legislation. Estimation of AFY is based on a multiplication of duration of employment (in years, assuming that a standard working years has 240 workdays), duration of performing asbestos-related tasks per day (in hours per day), and level of exposure defined as fibre concentration (in fibres per cubic centrimetres (F/cm^3^)). As explained in the example by Felten et al., a worker performing the task of spraying an asbestos pump with a fibre concentration of 400 F/cm^3^ for one standard working year of 240 workdays and 8 h per workday would accumulate 400 AFY [25].

**Table 1:** Occupations covered by the job-specific supplementary questionnaires

Occupation	
Agriculture and forestry, horticulture, fruit, wine, tobacco, hops and vegetables cultivation, road workers, train maintenance	Painter, varnisher and house painter
Roof tiler and façade builder	Woodworking and processing, furniture production, parquet production (carpenters, forestry and sawing workers, track-builders, staircase builders, parquet layers, chipboard and plywood production)
Insulation worker	Ventilation technician, air conditioning technician, pipe network builder
Electrician and control technician	Roadworks and civil engineering workers
Stonemason and stone worker terrazzo dealers and manufacturers	Metal production worker
Metalworking and processing	Foundry
Heat protection	Welding, flame cutting, flame spraying
Motorcar mechanic	Locksmith, plumber and installer
Asbestos processing industry	Chemical / pharmaceutical industry, mineral mil processing, fertiliser production
Electroplating	Leather production or processing, tannery
Cokery, gas plant	Nuclear industry
Health services	Mining
Rubber industry	Textile industry
Air and aerospace industry	Protection of goods in stock, fumigation facilities, sterilisation plant, grain and food wholesalers, mill operations
Warehouse worker, Stevedore dock worker	Shipyard workers, shipbuilders
Building construction, masons, plasterers, stuccoers	Soldering
Diner, cook, kitchen help	Not unambiguously classifiable

### Potential confounders

The study questionnaire also collected information on potential confounders starting with socio-demographic information (age, schooling, highest educational level) and current occupational status (still working full time, part time, or retirement). In addition, the questionnaire asked for information on the participants’ health and lifestyle with items being based on questionnaires from previous studies such as the European Community Respiratory Health Survey [26]. The participants were also asked if there were cases of cancer in their family (i.e., parents and siblings), and if the answer was affirmative, more details on the type of cancer diagnosed and the dates of diagnosis were collected. Furthermore, they were inquired about personal history of ovarian cancer, any other cancer, and if any surgery was performed on their abdomen. In the lifestyle part of the questionnaire, participants were questioned about the use of talcum powder and duration of its use in genital area or other body parts. The lifestyle part also included questions regarding ever smoking (defined as a minimum of 20 packets of cigarettes during lifetime, or at least one cigarette per day for 1 year), physical activity (not active: mostly sitting throughout work; moderately active: being active for 20 min three times a week over a period of 10 years; highly active: doing sports every week for 30 min at least three times per week), number of pregnancies, birth year of the offspring, age at menarche and menopause, and breastfeeding (yes/no) including duration of breastfeeding. An English translation of the study questionnaire is provided in the supplement (Additional file 1).

### Collection of medical documentation

We collected medical documentation including records from imaging procedures (CT, MRI and x-ray scans) from all interviewees, where available. The images were then screened for indication of pleural plaques by two independent experienced radiologists/occupational physicians (KGH and TK). In addition, we aimed at retrieving histological materials from the suspected ovarian cancer cases for tumour validation by a pathologist specialised in diagnosis of asbestos-related carcinoma (AT). The documents were obtained from the interviewees themselves, from their treating doctors and hospitals, or from records available at the health service of the cooperating trade association.

### Feasibility criteria

We evaluated the feasibility of the planned, large-scale study by using the following, a priori defined criteria:

i) A response of at least 60%, to be able to detect a relative risk of 2 with 90% statistical power.
ii) A number of five cases (95% confidence interval: 2–11 cases) among 800 participants.
iii) Reliable and exact estimation of asbestos fibre-years for all suspected cases and at least 25% of all participants.
iv) Medical documentation (records from imaging procedures, medical reports, histological materials) from all cases and records from imaging procedures from at least 25% of the entire sample.

### Bias

We considered recall bias as a potentially significant source of bias due to difficulties of the individuals to correctly report asbestos exposure as well as start and end dates of their jobs. Therefore, when making an appointment with the participants the interviewers told them to put out ready any work contracts they had with them at the day of the interview in order to be able to look up for the details during the interview. Interviewer bias was addressed with professional training of the interviewers instructing them to conduct the interviews in a standardised form to reduce the influence on respondents’ answers. The interviewers also underwent asbestos-specific training by an expert at the German Social Accident Insurance.

### Statistical analysis

We assessed the feasibility criteria by calculating the following statistics:

i) Absolute number and percentage of individuals who participated in the study (response); as birth dates for all invitees were available, we additionally calculated if participants differed from non-participants with respect to age (arithmetical mean including 95% confidence interval and *p*-value from independent t-test for continuous variables).
ii) Absolute number of cases detected in the study; we additionally calculated the latency period (i.e., the time between the first reported occupational exposure to asbestos and the date of the relevant diagnosis) and the interim period (i.e., the time between the last reported occupational exposure to asbestos and the date of the relevant diagnosis).
iii) Absolute number and percentage of individuals whose self-reports regarding asbestos exposure where sufficient to allow reliable and exact estimation of fibre-years.
iv) Absolute number and percentage of individuals for whom we could obtain medical documentation.

Furthermore, we present summary statictics to describe the socio-demographic, lifestyle, and health-related characteristics of the study participants. Summary statistics were calculated as arithmetical means and standard deviations (SD) for continuous variables while categorical variables were expressed as absolute numbers (n) and percentages (%).

Statistical analyses were performed in R version 1.0.44 and SPSS Version 25.

## Results

### Feasibility criteria

#### Response

Out of 1.000 invitees, six persons could not be contacted due to an invalid address while 23 persons were reported to be deceased, leaving a net sample of 971 individuals. Among those, 204 agreed to participate, 411 gave a negative response, and 356 did not answer after the second reminder. Out of the 204 individuals giving informed consent, 163 were actually interviewed, while 41 individuals could not be reached for making an appointment or did not answer the phone at the agreed date of the interview. Hence, the response was in total 17%. Those 204 individuals who gave informed consent were on average 61.6 years old (95% CI: 60.3–62.9 years), while the 767 invitees who gave no or a negative answere had an average age of 66.3 years (95% CI: 65.5–67.1 years, *p* < .05).

Among the 163 interviewees, 119 gave their informed consent after the initial invitation letter, 32 after the first reminder and 12 after the second reminder.

#### Number of cases

Based on participants’ self-reports in the interview, a history of ovarian cancer was initially suspected in six individuals. In two interviewees, this suspicion was confirmed by the available medical documentation (one because of histology reports, one because of medical reports). The other tumour suspects could not be confirmed by the obtainable medical documents. Dates of the relevant diagnoses were between 1993 and 2008. The latency period was on average 28.7 years, while the interim period was on average 12.5 years. Applying the above-mentioned estimation of the expected number of cases (5 cases among 800 individuals) to the actual number of participants in the pilot study, which turned out to be only 163 participants, one case could be expected. Therefore, our observed number of tumour suspect cases is well within the expected number of cases.

#### Asbestos fibre-years estimations

Using the data from the job-specific supplementary questionnaires, it was possible to exactly calculate individual exposure in asbestos fibre-years for 48 of the 163 participants (29%). Among those individuals was, however, only one of the suspected cases. For an additional 96 interviewees (59%), at least categorical classification of fibre-years could be conducted (no exposure, < 3 AFY, 3 < 6 AFY, 6 < 9 AFY, 9 < 12 AFY, > 12 AFY).

Those 48 individuals with exact fibre-year estimation were exposed for an average of 5.6 AFY (SD: 10.4 AFY, Minimum: 0.0 AFY, Maximum: 64.8 AFY). Assigning those 48 subjects to the above-mentioned categories, a total of 144 subjects could be classified according to those categories. Most of the participants (29%) accumulated less than three fibre-years, whereas the highest exposure level of more than twelve fibre-years was observed only in 18 subjects (11%; Table 2). With respect to asbestos exposure levels in the suspected ovarian cancer cases, the two confirmed cases reported low exposure levels (no exposure and < 3 AFY, respectively), while among the four non-confirmed cases three were classified into the 3 < 6 AFY category and one into the 9 < 12 AFY category.

**Table 2:** Distribution of the participants with respect to exposure categories calculated in asbestos fibre-years (*N* = 144)

Exposure category	Absolute number (n)	Percentage (%)
No exposure	21	12.9
< 3 AFY	47	28.8
3 < 6 AFY	28	17.2
6 < 9 AFJ	20	12.3
9 < 12 AFY	10	6.1
≥12 AFY	18	11.0

*AFY* Asbestos fibre-years

#### Medical documentation

Medical documents including records from imaging procedures were obtained for a total of 79 individuals (8% of all invitees and 48% of all participants), among them four of the six tumour suspects. However, histologocial materials could not be retrieved for any of them; only for one suspected case we were able to get hold of the histology report. Records from imaging procedures that had sufficient quality for radiologic analysis with regard to screening of pleural plaques was available for twelve participants. In none of them, we observed indication for presence of pleural plaques.

Altogether, the pilot study fulfilled only one out of the four feasibility criteria (Table 3).

**Table 3:** Summary of the assessment of the feasibility criteria

Criterion	Absolute number (n)	Percentage (%)	Criterion fulfilled
Response	163	17%	No
Number of cases	6 suspect, 2 confirmed cases	4% / 1% of all participants	Yes
Exact estimation of asbestos fibre-years	1 of 6 suspect cases; 48 of 163 all participants	17% of cases; 29% of all participants	No
Medical documentation (including imaging procedures)	79 (12)	8% (.01%) of the entire sample 48% (7%) of all participants	No

### Descriptives

At the time of the interview, the 163 individuals were on average 63 years old. About half of the participants had medium schooling level, a quarter reported low educational level. When asked for their current occupation, about 50% of the individuals reported to be retired. The total number of self-reports of cancer diagnoses other than ovarian cancer was 28, which included breast (9), skin (4), bowel (6), peritoneal (1), splenic (1), thyroid (1), and cervical cancer (5) as well as brain tumour (1). The reported dates of diagnoses were between 1973 and 2017 with twelve reported diagnoses dating back more than 10 years. More than half (53%) of the participants had a family history of cancer and around 37% had a history of gynaecological problems. 146 subjects (89%) reported having ever worked with asbestos. With regard to gynaecologic aspects, individuals indicated an average of two pregnancies. Average duration of OCP use was 15 years. More than half of the subjects reported having had a lower abdomen surgey; in 36 interviewees (22%) this was a surgery of the ovaries. In all variables, the number of missing values was below 10 %, most of them were 100% complete (Table 4).

**Table 4:** Socio-demographic, occupational, health-related, and lifestyle characteristics of the participants (*N* = 163)

	Missing	Mean	SD
Age (years)	0	62.8	9.5
Age at menarche	6	13.6	1.5
Age at menopause	14	48.1	6.1
Number of pregnancies	0	2.1	0.9
Breastfeeding (months)	3	6.0	7.9
OCP use (years)	0	15.0	9.7
	Missing	N	%
Schooling^a^	0		
Low		41	25.1
Medium		81	49.6
High		41	25.1
Professional education^b^	0		
No		10	6.1
Medium		117	71.7
High		36	22.0
Current occupation	0		
Full time/part time		73	44.7
Retired		78	47.8
Other/Housewife/Job-seeking		12	7.3
Cancer history			
Ovarian cancer	0	6	3.6
Any other cancer	0	28	17.2
Family history of cancer	0	88	53.9
Gynaecological history	0	61	37.4
Ovarian inflammation		8	13.1
Ovarian cyst		13	21.3
Uterus myoma		13	21.3
Ectopic pregnancy		1	0.6
Other		26	42.6
Lower abdominal surgery	0	93	57.0
Ovarian surgery		36	22.1
Hysterectomy		26	15.9
Other		31	19.0
Physical activity^c^	5		
Not active		16	10.1
Medium		86	54.4
High		56	35.4
Smoking (ever)	0	75	46.0
Cosmetic powder use in genital area	0	1	0.6
Cosmetic powder use in other body parts	0	27	16.5

*SD* Standard deviation

^a^Schooling: low = primary/secondary moden school; medium = high-school diploma; high = university of applied sciences entrance qualification/A levels/technical diploma

^b^Professional education: no = no degree; medium = professional school/vocational school/apprenticeship; Hhgh = college/university

^c^Physical activity: not active = mostly sitting throughout work; moderately active: being active for 20 min three times a week over a period of 10; highly active = doing sports every week for 30 min at least three times per week

## Discussion

The aim of this pilot study was to evaluate the feasibility of a large-scale epidemiological study elucidating the association between asbestos exposure and ovarian cancer in a sample of 16.000 women registered as having been occupationally exposed to asbestos. To achieve this aim, we invited a sample of 1.000 of these women to participate in a pilot study. The feasibility of the planned main study was assessed on the basis of potential participants’ willingness to join the study, the number of identified cases of ovarian cancer, the viability of performing detailed estimation of asbestos exposure levels based on participants’ self-reports, and availability of medical documentation.

The results of the pilot study indicate that the feasibility of the planned main study is very limited. Especially the response of 17% was well below the target minimum of 60%. Also the availability of medical documentation was rather restricted. Although at least some documents could be collected for at least half of the participants, due to the low response those subjects constitute only 8 % of the entire sample. Moreover, we could not retrieve histological samples for tumour validation for any of the suspected cases of ovarian cancer. With respect to the criterium of detailed assement of exposure levels via estimation of asbestos fibre years, we could perform such estimation for the requested minimum of 25% of all participants, but only for one of the suspected cases. The only criterium that we fulfilled was the number of identified cases. Even though only two out of the six suspected cases could be confirmed by the obtainable medical documentation, this number is still within the confidence interval of expected cases. This is remarkable insofar as this confidence interval was originally calculated for 800 participants.

The weightiest aspect speaking against the feasibility of the planned main study is, however, the low response. This could be explained at least partly by the steadily decreasing willingness in the general population, especially also in Germany, to take part in scientific surveys [27, 28]. To receive reliable results that are not overly affected by selection bias [29], markedly higher response levels are necessary. The comparison of the age structure of the participants and non-participants, for instance, indicated that rather the younger invitees agreed to take part, pointing towards selection bias by age. The rather high average age of the invited sample might also be an explanation for the low response itself, as some invitees who gave their negative answers by calling at the study centre pointed out that they see low personal relevance in the study since their occupational exposure to asbestos dated back many years or decades. What concerns the personal relevance of the study for invitees, one could also assume that particularly those invitees that have been exposed for a very short period or to very low levels, had low interest to join the study. What speaks against this assumption though is the fact that among the majority of our participants we observed rather low exposure levels. This, on the other hand, could imply that especially those invitees that had high exposure levels in their past were not able any more to take part because of health-related restrictions. In this context, we need to highlight the limitation of our approach of not being able to collect data on women that already passed away. Therefore, survival bias is likely to have played a role in our study. This assumption is supported by the fact that in our study population we did not observe cases of lung cancer or pleural mesothelioma, two malignant diseases that are very well known to be associated with asbestos exposure. Here again, the most likely explanation for this lack is that those women who had such a diagnosis meanwhile passed away or where too sick to answer the questionnaire.

The large time lag is also the most likely explanation for the limited availability of medical documentation that could be retrieved. Specifically among the suspected cases of ovarian cancer, all relevant dates of diagnosis dated back at least 10 years ago. Accordingly, many of the doctors and hospitals where the respective women reported having been treated indicated that they have no or only very few documents anymore or that the subjects could not be identified anymore in their archives. Our initial aim of screening imaging records from the participants for pleural plaques thus seems to be hardly viable.

If the study is planned further, especially the recruitment of participants needs to be improved clearly. Inviting the entire cohort of all remaining 15.000 registered insurees appears unrealistic. Given the low validity of the results that can be expected because of the high likelihood of selection bias, the high amount of labour and financial costs to send out invitations to a five-digit number of individuals can hardly be justified.

An alternative study design may be an incident case-control study with rapid case ascertainment, as this would at least help to overcome the influence of survival bias. Nevertheless, also with this approach steps to increase the motivation of invitees to participate in the study need to be found. While it can be assumed that among cases the response should be relatively high due to the high personal meaning of such a study (an assumption that is supported by the relatively high number of tumour suspects among the few participants of the pilot study), especially intense efforts for recruitment and motivation of controls would be necessary. On the one hand, these could be established by use of methods such as financial incentives, while on the other hand measures could be taken to raise the personal relevance of the study for controls, for example hand-written envelopes or first contact via phone to explain the importance of the study in person. In addition, the hurdle to participate might be lowered by deleting some of the potential confounders that were hardly reported by the participants of the pilot study (such as use of talcum powder in the genital area) as to reduce the length of the questionnaire. All these measures have been shown to be effective in previous studies or in systematic reviews that especially explored methods to increase response in epidemiological studies [30–32].

Lastly, the present study has some strengths that should be mentioned. This was the first ever study to be conducted in Germany to explore not only the feasibility of a large study on specifically investigating the association between asbestos exposure and ovarian cancer, but also aiming to investigate the dose-response relationship between the two. Moreover, this study is based on a large database of asbestos-exposed women, which are hard to reach in the general German population at present due to the ban on asbestos since 1993. Moreover, this pilot study brings valuable information regarding effective use of resources for further planning not only of this project but also of similar studies. For example, one could conclude that even though occupational registers may be a useful starting point for cohort studies, they may find their limits with respect to ascertainment of cases, especially in retrospective cohort studies. Particularly for diseases with high mortality, incident case-control studies with rapid recruitment of cases as soon as possible after diagnosis may be the more viable option. Our results may thus provide helpful information for the planning of other studies, not only in the field of ovarian cancer but also of other fatal cancer types.

## Conclusions

To conclude, the results of the present pilot study indicate that the feasibility of the study design for a large retrospective cohort study is very limited, mainly due to low response and restricted availability of medical documents. Hence, for further planning, ways to increase response and to overcome survival bias need to be found.

## Supplementary information

**Additional file 1.** Study questionnaire. English translation of the study questionnaire.

## Data Availability

The datasets used and/or analysed during the current study are available from the corresponding author on reasonable request.

## Abbreviations

AFY: Asbestos fibre-years
BRCA 1 and BRCA 2: Breast cancer genes 1 and 2
OCP: Oral contraceptive pills
SD: Standard deviation
