# Supplementary material for: Investigating the association between occupational exposure to asbestos and ovarian carcinoma: results from a pilot study in Germany
Source: BMC Public Health. 2019 Oct 22;19:1341. doi: 10.1186/s12889-019-7590-7 (PMC6805637; doi:10.1186/s12889-019-7590-7)
Supplement: Supplementary file 1 — Additional file 1. Study questionnaire. English translation of the study questionnaire. [file 12889_2019_7590_MOESM1_ESM.pdf]

LMU
KLINIKUM
DER UNIVERSITÄT MÜNCHEN

ARBEITSGRUPPE ARBEITS- UND
UMWELTEPIDEMIOLOGIE & NET TEACHING
LEITUNG: PROF. DR. KATJA RADON, MSC
INSTITUT UND POLIKLINIK FÜR
ARBEITS-, SOZIAL- U. UMWELTMEDIZIN
DIREKTOR: PROF. DR. MED. DENNIS NOWAK
CAMPUS INNENSTADT

Study Questionnaire

Study ID: ______

GENERAL INFORMATION

First, we would like to ask you for some general information about yourself.

1 What is your birth date?

I__I__I.I__I__I.19I__I__I
Day Month Year

2 Which is your highest level of schooling?

- Primary/secondary modern school qualification .... ☐
- High-school diploma .... ☐
- University of applied sciences entrance qualification .... ☐
- A levels .... ☐
- Technical diploma... ☐
- Other,... ☐

Please specify ____________________________________

3 Which is your highest vocational qualification?

- Professional school/vocational school................ ☐
- Apprenticeship .... ☐
- College/University .... ☐
- No degree.. ☐

Other, ... ☐

Please specify:

____

4 Which is your current occupation?

Working full-time .... ☐

Working part-time .... ☐

Retired.. ☐

Housewife .... ☐

Job-seeking .... ☐

Other, ... ☐

Please specify:

____

OCCUPATIONAL HISTORY

We would now like to ask you about **all the professional activities** that you have had so far. By profession, we mean the activity with which you have spent most of your time. Please consider all activities that you have performed **for at least 12 months**. Please start with your current or last performed professional activity.

5 First (=current/last) activity

5.1 Start date: I__I__I__I__I/I__I__I
Year Month

End date: I__I__I__I__I/I__I__I
Year Month

(if your are still working in this job, please indicate the current date)

5.2 What is the name of the institution/company?

____

5.3 Which task do/did you perform?

____

5.4 Did you work full-time, part-time or occasionally in this job?

I__I Full-time (100%)
I__I Part-time (e.g., 75 or 50%)
I__I Occasionally (less than 50%)

5.5 Can you remember contact with asbestos or asbestos-containing products / work equipment during this activity?

No .... ☐
Yes .... ☐

6 Second activity

6.1 Start date: |__|__|__|__|/|__|__|
Year Month

End date: |__|__|__|__|/|__|__|
Year Month

6.2 What was the name of the institution/company?

6.3 Which task do/did you perform?

6.4 Did you work full-time, part-time or occasionally in this job?
|__| Full-time (100%)
|__| Part-time (e.g., 75 or 50%)
|__| Occasionally (less than 50%)

6.5 Can you remember contact with asbestos or asbestos-containing products / work equipment during this activity?
No .... ☐
Yes .... ☐

7 Third activity

7.1 Start date: I__I__I__I__I/I__I__I
Year Month

End date: I__I__I__I__I/I__I__I
Year Month

7.2 What was the name of the institution/company?

____

7.3 Which task do/did you perform?

____

7.4 Did you work full-time, part-time or occasionally in this job?

I__I Full-time (100%)
I__I Part-time (e.g., 75 or 50%)
I__I Occasionally (less than 50%)

7.5 Can you remember contact with asbestos or asbestos-containing products / work equipment during this activity?

No .... ☐
Yes .... ☐

8 Fourth activity

8.1 Start date: I__I__I__I__I/I__I__I
Year Month

End date: I__I__I__I__I/I__I__I
Year Month

8.2 What was the name of the institution/company?

8.3 Which task do/did you perform?

8.4 Did you work full-time, part-time or occasionally in this job?

I__I Full-time (100%)
I__I Part-time (e.g., 75 or 50%)
I__I Occasionally (less than 50%)

8.5 Can you remember contact with asbestos or asbestos-containing products / work equipment during this activity?

No .... ☐
Yes .... ☐

9 Fifth activity

9.1 Start date: I__I__I__I__I/I__I__I
Year Month

End date: I__I__I__I__I/I__I__I
Year Month

9.2 What was the name of the institution/company?

____

9.3 Which task do/did you perform?

____

9.4 Did you work full-time, part-time or occasionally in this job?

I__I Full-time (100%)
I__I Part-time (e.g,. 75 or 50%)
I__I Occasionally (less than 50%)

9.5 Can you remember contact with asbestos or asbestos-containing products / work equipment during this activity?

No .... ☐
Yes .... ☐

HEALTH

Now we would like to ask you about your health. If you are not sure about any of the questions, please answer "No".

10 Have you ever been diagnosed with ovarian cancer?

No .... ☐

Yes .... ☐

If yes, when was it first detected?

|__|__|__|__| Year

11 Have you ever been diagnosed with <u>another type of cancer</u>?

No .... ☐

Yes .... ☐

If yes, which type of cancer?

____

If yes, when was it first detected?

|__|__|__|__| Year

12 Has your family (that is, your parents or siblings) ever been diagnosed with cancer?
If so, who was affected, what type of cancer was it, and when was it diagnosed?

Mother................................ ☐ (Type:______________________________; Year of diagnosis:__________)

Father.............................. ☐ (Type:____________________________________; Year of diagnosis:___________)

Sibling 1.......................... ☐ (Type:____________________________________; Year of diagnosis:___________)

Sibling 2.......................... ☐ (Type:____________________________________; Year of diagnosis:___________)

Sibling 3.......................... ☐ (Type:____________________________________; Year of diagnosis:___________)

Sibling 4.......................... ☐ (Type:____________________________________; Year of diagnosis:___________)

13 Have you ever been diagnosed with gynaecological disease (such as endometriosis, inflammation of the fallopian tubes, polycystic ovarian syndrome)?

No .... ☐

Yes .... ☐

If yes, which type of disease?

____

When was it detected?

|__|__|__|__| year

14 Have you ever undergone abdominal surgery (such as surgical removal or fallopian tube obstruction)?

No .... ☐

Yes .... ☐

If yes, which type of surgery?

____

When was the surgery performed?

|__|__|__|__| Year

LIFESTYLE

15 Which statement regarding your physical activity is true for you in the past?

I had a sedentary job and was not active……………………..…………☐

I was active 3 times a week for 20 min over a period of 10 years…….☐

I did sports every week for at least 3 times a week for 30 minutes over a period of 10 or more years ..……………..……….....................☐

16 Have you ever smoked for a year?

"Yes" means at least 20 packs of cigarettes or 360g of tobacco in your life or one year a day or a cigar a week

No.. ☐

Yes... ☐

17 Have you ever regularly (i.e., at least once a month) used cosmetic powder (talcum powder) in the genital area (for example on sanitary towels, tampons, etc.)?

No.. ☐

Yes... ☐

17.1 If yes, from when to when did you use cosmetic powder in the genital area?

From: |__|__|__|__| to: |__|__|__|__|
Year Year

18 Have you ever regularly (i.e., once a month) used cosmetic powder (talcum powder) outside the genital area (for example, when powdering your baby or on your own feet or arms or face)?

No .... ☐

Yes .... ☐

18.1 If yes, from when to when did you use cosmetic powder outside the genital area?

From: |__|__|__|__| to: |__|__|__|__|
Year Year

19 Have you ever received hormone replacement therapy?

No .... ☐

Yes .... ☐

19.1 If yes, from when to when did you receive hormone replacement therapy?

From: |__|__|__|__| to: |__|__|__|__|
Year Year

20 Have you ever taken the "pill" (oral contraceptives)?

No .... ☐

Yes .... ☐

20.1 If so, how many years did you take the "pill" altogether?

______ years

21 At what age did you get your first menstrual period?

Age: |__|__| years

22 At what age did you get your last menstrual period?

Age: |__|__| years

23 Did you have any pregnancies (including aborted pregnancies and miscarriages)?

No .... ☐

Yes .... ☐

23.1 If yes, how many pregnancies did you have?

______ pregnancies

23.2 In which year were your children born?

First child: |__|__|__|__| Year

Second child: |__|__|__|__| Year

Third child: |__|__|__|__| Year

Fourth child: |__|__|__|__| Year

Fifth child: |__|__|__|__| Year

23.3 Have you breastfed your children?

No .... ☐

Yes .... ☐

23.4. If yes, how many years have you breastfed in total?

______ Year

Thank you very much for your participation!

Do you have comments on this questionnaire? We are grateful for suggestions!

____

____

____

____
